# Effectiveness of Telehealth Versus In-Person Informed Consent: Randomized Study of Comprehension and Decision-Making

**DOI:** 10.2196/63473

**Published:** 2025-03-05

**Authors:** Saif Khairat, Paige Ottmar, Prabal Chourasia, Jihad Obeid

**Affiliations:** 1 Carolina Health Informatics Program University of North Carolina at Chapel Hill Chapel Hill, NC United States; 2 Lineberger Comprehensive Cancer Center University of North Carolina at Chapel Hill Chapel Hill, NC United States; 3 Cecil G Sheps Center for Health Services Research University of North Carolina at Chapel Hill Chapel Hill, NC United States; 4 School of Nursing University of North Carolina at Chapel Hill Chapel Hill United States; 5 Medical University of South Carolina Charleston, SC United States

**Keywords:** telehealth, informed, consent, comprehension, decision-making, cross-sectional study, cross-section, telemedicine, eHealth, health care services, mHealth, effectiveness, informed consent, statistical analysis, feasibility

## Abstract

**Background:**

Obtaining informed consent (IC) is vital for ethically and effectively recruiting participants in research projects. However, traditional in-person IC approaches encounter notable obstacles, such as geographic barriers, transportation expenses, and literacy challenges, which can lead to delays in enrollment and increased costs. Telehealth, especially teleconsent, offers a potential way to overcome these obstacles by facilitating the IC process in a digital setting. Nonetheless, there are concerns about whether teleconsent can achieve levels of understanding and involvement that are equivalent to those of in-person IC meetings.

**Objective:**

This study aims to evaluate comprehension and decision-making in participants undergoing teleconsent versus traditional in-person IC. We used validated assessments to determine whether teleconsent is a viable alternative that maintains participants’ understanding and decision-making abilities.

**Methods:**

A randomized comparative study design was used, recruiting potential participants for a parent study assessing patient experiences with patient portals. Participants were randomly assigned to 2 groups: teleconsent and in-person consent. The teleconsent group used Doxy.me software, allowing real-time interaction between researchers and participants while reviewing and electronically signing the IC documents. Recruitment involved using an institutional web-based platform to identify interested individuals, who were then contacted to assess eligibility and gather demographic information. The Decision-Making Control Instrument (DMCI) survey was used to assess the perceived voluntariness, trust, and decision self-efficacy. The Quality of Informed Consent (QuIC) was used to measure the comprehension level of the consent form. The validated Short Assessment of Health Literacy-English tool was used to measure participants' health literacy levels.

**Results:**

A total of 64 participants were enrolled in the study, with 32 in the teleconsent group and 32 in the in-person group. Of 64 participants, 32 (50%) were in the teleconsent group, 54 (84.4%) were females, 44 (68.7%) were aged 18-34 years, 50 (78.1%) were White, and 31 (48.4%) had a bachelor degree. The mean SAHL-E scores were different between the teleconsent and in-person groups (16.72, SD 1.88 vs 17.38, SD 0.95; *P*=.03). No significant differences were found between the average scores at baseline and follow-up for QuIC part A (*P*=.29), QuIC part B (*P*=.25), and DMCI (*P*=.38) within the teleconsent and in-person groups. Additionally, there were no significant differences in QuIC or DMCI between subgroups based on age, sex, and ethnicity.

**Conclusions:**

This study assessed the effectiveness of IC processes through telehealth compared to traditional in-person visits. Findings indicate that telehealth offers similar participant understanding and engagement while overcoming geographic and accessibility barriers. As health care adopts digital solutions, these results highlight telehealth’s potential to improve recruitment and retention in clinical research, suggesting that policy makers should integrate telehealth practices into regulations for better access and health outcomes.

## Introduction

Obtaining informed consent (IC) supports the successful recruitment of participants and the overall success of research studies [1]. However, obtaining IC faces challenges related to the cost and time to travel to and from the study site, geographic barriers in rural or remote areas, and literacy barriers [2]. Lack of obtaining IC leads to delayed enrollment, low retention, and engagement rates, and increased costs for additional resources [3].

Traditional methods of obtaining IC usually involve face-to-face interactions in which health care providers explain procedures, risks, benefits, and alternatives to patients, often using written consent forms [4,5]. These methods facilitate direct communication and allow patients to ask questions, which helps build understanding and trust [5,6]. However, several challenges are associated with this process. Patients often have varying levels of health literacy, and time constraints can prevent thorough discussions [7,8]. Additionally, the use of complex medical jargon can confuse patients [9,10].

Research has shown that many patients are unaware of their rights and may not receive enough information to make fully informed decisions [11,12]. Challenges in decision-making and comprehension of in-person IC are multifaceted. Patients often have varying levels of health literacy, which can significantly impact their understanding of medical information and their ability to make informed decisions [13]. Time constraints during consultations can further hinder thorough discussions, leaving patients with insufficient information [7]. Additionally, the use of complex medical jargon can confuse patients, making it difficult for them to grasp the details of their treatment options [14]. Research has shown that many patients are unaware of their rights and may not receive enough information to make fully informed decisions [11]. Furthermore, the responsibility for obtaining consent can sometimes be unclear, leading to inconsistencies in the process [15,16]. To address these challenges, it is essential to improve communication strategies and ensure that consent forms are clear and comprehensive.

The use of telehealth, namely teleconsent, has the potential to address these challenges by conducting the IC process digitally [17]. Teleconsent involves live video calls during which the researcher and participant can see and interact with each other in real-time [18,19]. There are concerns about the ability of teleconsent to deliver similar levels of comprehension and engagement to in-person IC visits. This noninferiority study aimed to examine the comprehension and decision-making levels for teleconsent versus in-person IC visits using validated comprehension assessment survey instruments in conjunction with health literacy evaluation.

## Methods

### Study Design

In this randomized comparative study, we obtained the IC of potential participants for a parent study that assesses patients’ experience using patient portals. Participants were recruited through an institutional web-based recruitment platform or word of mouth. Participants were randomly assigned to in-person or teleconsent. The teleconsent group used Doxy.me software, which enables the researcher to share the IC document on-screen, collaboratively complete it with the participant and sign it electronically. Individuals in the in-person group met with the study research assistant in a private office.

### Recruitment Process

We listed the study on an institutional recruitment platform such that when an individual expressed interest in the study, the research assistant received an automated notification email containing the potential participant's contact information.

The research assistant had initiated contact via email acknowledging the individual’s interest and requesting demographic and eligibility information to ensure participant diversity. The research assistant provided a link to a Qualtrics survey and asked qualified individuals to digitally sign a consent form. The research assistant emphasized confidentiality and mentioned an upcoming interview, which could be in person or via teleconsent. Participants would also receive a gift card for their involvement in both the initial interview and a follow-up session.

Participants designated for teleconsent received an email from the research assistant confirming their eligibility for remote participation via computer (microphone and camera required; smartphones and tablets not allowed). They were asked to complete a scheduling form for a 45-minute session, which included the consent process, a semistructured interview on MyChart usability, and follow-up questionnaires. The research assistant also mentioned a gift card incentive for both initial and follow-up sessions.

In cases where participants were scheduled for an in-person session, the research assistant issued a similarly structured email. The message confirmed their eligibility and presented them with the option to choose between 2 locations: a public library or a private office at the University. Participants were encouraged to fill out a scheduling form with their preferred time slots. The research assistant outlined the session’s duration and activities, mirroring the information provided for teleconsent, along with the mention of the gift card reward for study completion.

### Consent Process

The consent form was 6 pages long, and it outlined the purpose, procedures, risks, and participant rights related to the study. The consent form was designed to inform potential participants about the study, ensuring they can make an informed decision about their involvement. It invited them to ask questions and clarified their rights as research subjects.

This consent form explained the nature of the study to evaluate participants’ perceptions of the MyChart patient portal’s usability and differences in consent understanding between teleconsent and traditional methods. The participants were adults who were users of MyChart, a prominent web-based patient portal. Participation in this study was voluntary. The form explained the chronological order of events in this study and that it will begin with eligibility questions and the collection of demographic information. Participants will then be randomly assigned to either teleconsent or in-person consent. Following this, they will take part in an interview with a research assistant regarding their experiences with MyChart, which will be recorded for transcription purposes. Additionally, participants will complete questionnaires on consent comprehension and health literacy.

The form also stated that the study will last no more than 1 hour and will be conducted at a designated location either in person or via videoconference. The research assistant also read the section about the potential risks, including confidentiality breaches, which will be minimized by assigning codes to data and ensuring anonymity during analysis. Finally, the form covers that the study is protected by a Certificate of Confidentiality, which prevents the use of identifying information in legal scenarios without consent, with some exceptions such as mandatory reporting laws. To verify the identity of the individuals in the teleconsent group, we asked that they enable their cameras during the entirety of the session. In addition, when signing the consent form, we used a feature in Doxy.me that enables taking a screenshot along with a timestamp to accompany the individual’s wet signature obtained live during the session. This feature ensures that the individual who signed the consent form is the intended individual, a necessary step in teleconsent [20].

After the session, participants completed the Decision-Making Control Instrument (DMCI) and Quality of Informed Consent (QuIC) surveys after the consent session (ie, baseline) and 30 days after the session (ie, follow-up). Data collection occurred between April 30, 2019, and June 29, 2021. The institution review board approval was obtained.

We used the DMCI, a 15-item validated instrument, to assess participants’ perceived voluntariness, trust, and decision self-efficacy in the consent form [21]. The DMCI assesses participants’ feelings of influence over decisions and their confidence in the fairness and transparency of the process. The maximum possible score is 30 and higher scores indicate greater perceived autonomy and trust in decision-making.

We used the QuIC to measure comprehension of the parent study consent form [22]. The QuIC consists of 2 parts: part A (14 items) assesses objective knowledge of study details, testing participants’ factual understanding of the consent process; and part B (6 items) measures perceived understanding, evaluating how confident participants feel about comprehending the consent materials. Together, the QuIC thoroughly evaluates both actual and perceived IC quality. The maximum possible score is 80 and higher scores reflect better comprehension and confidence, ensuring that consent is truly informed.

We used the validated Short Assessment of Health Literacy-English (SAHL-E) tool to measure participants’ health literacy levels. The SAHL-E is an 18-item tool that assesses functional health literacy by testing participants’ ability to understand and apply health-related terms. It uses a word association format to evaluate vocabulary and conceptual knowledge. The maximum possible score is 18, and higher scores indicate better health literacy, which correlates with improved capacity to make informed health decisions (Multimedia Appendix 1).

### Ethical Considerations

The University of North Carolina at Chapel Hill institutional review board deemed this study exempt (17-2870). Participants consented to participate in the study and to be assigned to either the control or intervention groups. Participant responses were kept anonymous, and participants were only referred to by subject ID. All data were kept separate from names and housed behind a secure, Health Insurance Portability and Accountability Act (HIPAA)–compliant server. Participants received a US $15 gift card upon completing the baseline surveys and another US $15 gift card upon completing the follow-up surveys.

### Data Analysis

We compared the demographic distribution of participants regarding sex, age, race, education, and income between teleconsent and in-person groups. We used a 2-sample, 2-tailed *t* test assuming unequal variances to compare the SAHL-E scores between the teleconsent and in-person groups to compare literacy levels. We compared the scores for the DMCI and QuIC surveys for both the teleconsent and in-person groups between baseline and follow-up using 2-sample *t* tests. We used a 2-sample, 2-tailed *t* test assuming unequal variances to compare the DMCI and QuIC score for subgroups on sex (male and female), age (18-34 years, 35-50 years, 51-64 years, and 65 years and older), and race (Asian, Black or African American, White, and mixed race).

We aimed to enroll 64 participants to provide 80% power to detect modest differences (effect sizes approximately 0.50) between groups with respect to the survey instrument scores. For example, we expect to be able to detect a 3.5-unit difference on the DMCI composite score (which has been reported to have a mean of 46.1, SD 7.0) and a 4.7-unit difference on the QuIC (which has been reported to have a mean of 77.8, SD 9.4).

## Results

### Overview

Of 64 participants, 32 (50%) were in the teleconsent group, 54 (84%) were females, 44 (68%) were aged 18-34 years, 50 (78%) were White, and 31 (48%) had a bachelor degree. The mean SAHL-E scores were different between the teleconsent and in-person groups (16.72, SD 1.88 vs 17.38, SD 0.95; *P*=.03; Table 1).

**Figure 1:**
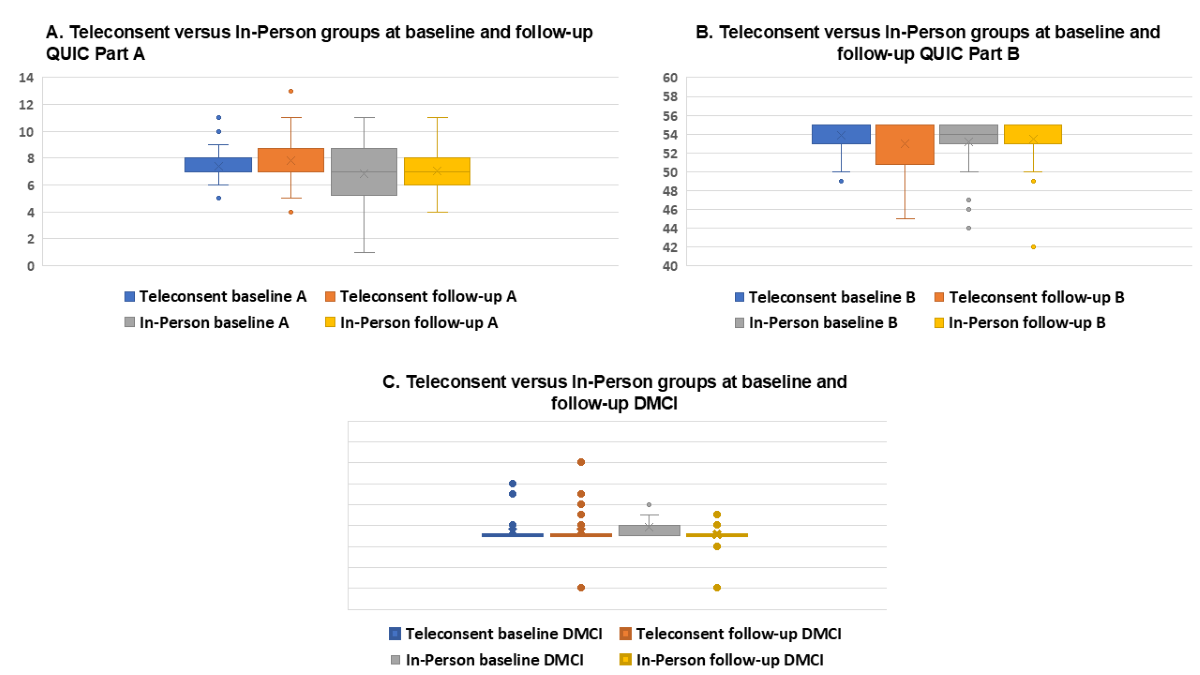
Average scores for (A,B) QuIC: (A) part A (maximum possible score=24) and (B) Part B (maximum possible score=56); and (C) DMCI (maximum possible score=30) at baseline and follow-up for the teleconsent and in-person groups. DMCI: Decision-Making Control Instrument; QuIC: Quality of Informed Consent.

At baseline, the teleconsent group had a higher mean QuIC score than the in-person group for part A (7.38, SD 1.27 vs 6.84, SD 2.46; *P*=.29) and part B (53.88, SD 1.80 vs 53.19, SD 2.79; *P*=.25). Both groups had similar mean DMCI scores (19.44, SD 1.12 vs 19.84, SD 2.31; *P*=.38; Figure 1).

**Table 1:** Demographic characteristics and health literacy scores of participants in teleconsent and in-person consent groups.

Category				All		Teleconsent (n=32)			In-person (n=32)			*P* value		
**Sex, n (%)**													.49	
		Male	10 (15)				4 (12)			6 (18)				
		Female	54 (84)				28 (87)			26 (81)				
**Age (years), n (%)**													.22	
	18-34		44 (68)		23 (71)			21 (65)						
	35-50		15 (23)		7 (21)			8 (25)						
	51-64		4 (6)		2 (6)			2 (6)						
	65 and older		1 (1)		0 (0)			1 (3)						
**Race, n (%)**													.40	
	American Indian		2 (3.1)		1 (3)			1 (3)						
	Asian		6 (9)		3 (9)			3 (9)						
	Black or African American		5 (7)		2 (6)			3 (9)						
	White		50 (78)		25 (78)			25 (78)						
	White Asian		1 (1)		1 (3)			0 (0)						
**Education, n (%)**													.08	
	Bachelor degree in college (4 years)		31 (48)		14 (43)			17 (53)						
	Master degree		13 (20)		6 (18)			7 (21)						
	Associate degree		5 (7)		3 (9)			2 (6)						
	Professional or doctoral degree		4 (6)		0 (0)			4 (12)						
	Some college but no degree		10 (15)		8 (25)			2 (6)						
	High school graduate		1 (1)		1 (3)			0 (0)						
**Income (US $), n (%)**													<.001	
	Less than 10,000		4 (6)		4 (12)			0 (0)						
	10,000-49,999		23 (35)		17 (53)			6 (18)						
	50,000-99,000		21 (32)		8 (25)			13 (40)						
	100,000-149,999		12 (18)		3 (9)			9 (28)						
	150,000 and more		4 (6)		0 (0)			4 (12)						
SAHL-E^a^				17.05		16.71			17.37			.03		

^a^SAHL-E: Short Assessment of Health Literacy-English.

At follow-up, the teleconsent group had a higher mean score than the in-person for the QuIC part A (7.84, SD 1.99 vs 7.03, SD 1.61; *P*=.08) and the DMCI (19.41, SD 1.77 vs 19.09, SD 1.13; *P*=.41). In-person had a higher mean score for the QuIC part B (52.94, SD 3.03 vs 53.50, SD 2.68; *P*=.44).

No significant differences were found between the average scores at baseline and follow-up for QuIC part A, QuIC part B, and DMCI within the teleconsent and in-person groups (Multimedia Appendix 2).

Within the teleconsent group, scores for QuIC part A increased from a baseline average of 7.38 (SD 1.27) to 7.84 (SD 1.99), while QuIC part B showed a slight decrease from 53.88 (SD 1.80) to 52.94 (SD 3.03). The mean DMCI scores remained relatively stable, with a minor decrease from 19.44 (SD 1.12) to 19.41 (SD 1.77). However, none of these changes were statistically significant.

Within the in-person group, the mean QuIC part A scores increased slightly from 6.84 (SD 2.46) to 7.03 (SD 1.61), and QuIC part B scores rose marginally from 53.19 (SD 2.79) to 53.50 (SD 2.68). The mean DMCI scores saw a notable drop from 19.84 (SD 2.31) to 19.09 (SD 1.13). Similar to the teleconsent group, none of these differences reached statistical significance.

### DMIC Subgroup Analysis

The results of the teleconsent and in-person consent assessments, measured by the DMCI scores, showed no significant differences, Table 2. However, males exhibited higher baseline scores in the teleconsent group (mean 20.25, SD 1.64) compared to females (mean 19.32, SD 0.97), although both groups showed similar follow-up scores. In-person assessments also indicated male scores were slightly higher at baseline (mean 19.50, SD 0.5) compared to females (mean 19.92, SD 2.54), but follow-up scores highlighted a more significant disparity between the groups.

**Table 2:** Comparison of DMCI^a^ scores by sex, age, and race for teleconsent and in-person consent groups at baseline and follow-up (maximum possible sore=30).

			Teleconsent								In-person						
			Baseline DMCI			Follow-up DMCI		*P* value		Baseline DMCI			Follow-up DMCI			*P* value	
**Sex, mean (SD)**																	
	Male	20.25 (1.64)			20.50 (1.12)		.83		19.50 (0.5)			19.33 (0.94)			.73		
	Female	19.32 (0.97)			19.25 (1.79)		.85		19.92 (2.54)			19.04 (1.16)			.12		
**Age (years), mean (SD)**																	
	18-34	19.52 (1.28)			19.17 (0.64)		.26		20.28 (2.75)			19.09 (1.38)			.09		
	35-50	19.14 (0.35)			20.14 (3.48)		.51		19 (0)			19.12 (0.33)			.35		
	51-64	19.5 (0.5)			19.5 (0.5)		≥.99		19 (0)			19 (0)			N/A^b^		
	65 and older	0 (0)			0 (0)		N/A		19 (0)			19 (0)			N/A		
**Race, mean (SD)**																	
	Asian	20.33 (1.89)			20 (1.41)		.85		24.33 (5.44)			19.66 (0.94)			.35		
	Black or African American	19 (0)			19.4 (0.8)		.37		19 (0)			19.5 (0.5)			.50		
	White	22 (2)			21 (2)		.75		19.5 (0.5)			19 (0)			.50		
	Mixed race	19.18 (0.39)			19.18 (1.85)		≥.99		19.4 (0.75)			19 (1.2)			.17		

^a^DMCI: Decision-Making Control Instrument.

^b^N/A: not applicable.

Age-related trends showed that younger participants (aged 18-34 years) had DMCI scores of 19.52 (SD 1.28) at baseline and 19.17 (SD 0.64) at follow-up via teleconsent, which were not significantly different (*P*=.26). Comparatively, older age groups showed varied results, with the 35- to 50-year-olds' scores being 19.14 (SD 0.35) and 20.14 (SD 3.48) for teleconsent, yielding a *P* value of .51. The 51- to 64-year age group had stable scores of 19.5 (SD 0.5) across both assessments, while there were no participants aged 65 years and older.

Racial comparisons show that Asian participants recorded the highest baseline score in the teleconsent group (mean 20.33, SD 1.89), while White participants presented more stable averages across both modes of consent. Asian participants had mean (SD) DMCI scores of 20.33 (1.89) and 20.00 (1.41) at baseline and follow-up via teleconsent (*P*=.85). Notably, the performance of Black or African American and mixed-race individuals illustrated lesser variability in scores.

### QuIC Subgroup Analysis

There were no significant differences in comprehension scores between teleconsent and in-person methods across the sex categories, with males reporting a baseline mean (SD) score of 61.25 (1.92) for teleconsent and 60 for in-person consent, while females scored 61.25 (2.18) and 60.04, respectively (Table 3). However, the *P* values for both sexes were .85 and .36, indicating no statistically significant differences between baseline and follow-up.

**Table 3:** Comparison of QuIC^a^ scores by sex, age, and race for teleconsent and in-person consent groups at baseline and follow-up (maximum possible sore=80).

			Teleconsent								In-Person						
			Baseline QuIC			Follow-up QuIC		*P* value		Baseline QuIC			Follow-up QuIC			*P* value	
**Sex, mean (SD)**																	
	Male	61.25 (1.92)			60.75 (4.09)		.85		60 (3.74)			59.33 (5.12)			.82		
	Female	61.25 (2.18)			60.79 (4.21)		.61		60.04 (3.17)			60.81 (2.70)			.36		
**Age (years), mean (SD)**																	
	18-34	61.30 (1.78)			61.78 (3.89)		.60		60.24 (3.28)			60.43 (3.92)			.87		
	35-50	61.14 (3.23)			58.86 (4.23)		.21		59.88 (2.76)			60.88 (1.76)			.43		
	51-64	61 (1)			58 (2)		.41		62 (0)			61.5 (0.5)			.50		
	65 and older	0 (0)			0 (0)		N/A^b^		53 (0)			58 (0)			N/A		
**Race, mean (SD)**																	
	Asian	61.66 (1.25)			60 (4.08)		.64		60 (3.56)			63.33 (0.47)			.32		
	Black or African American	62.2 (0.98)			59.4 (4.08)		.25		62.5 (0.5)			61.5 (0.5)			.29		
	White	61.05 (2.33)			61.27 (3.60)		.81		59.92 (3.31)			60.28 (3.45)			.71		
	Mixed race	60.5 (2.5)			60 (8)		.96		59 (3)			58.5 (3.5)			.92		

^a^QuIC: Quality of Informed Consent.

^b^N/A: not applicable.

The 18-34 age group had a teleconsent baseline mean (SD) score of 61.30 (1.78) compared to 60.24 (3.28) for in-person, with a *P* value of .87 indicating no significant difference. The 35-50 age group’s scores exhibited a teleconsent baseline of 61.14 (3.23) and an in-person score of 59.88 (2.76), with a *P* value of .43, again highlighting a lack of significant differences. The older age groups (51-64 years) demonstrated teleconsent baseline scores of 61 (SD 1) and 58 (SD 2) respectively for in-person, yielding a *P* value of .41.

Regarding race, the Black or African American group exhibited the highest baseline teleconsent score of 62.20 (SD 0.98), while the lowest was seen in the mixed-race category, scoring 60.50 (SD 2.50). Mixed race individuals exhibited a teleconsent score of 60.5 (SD 2.5) and 59 (SD 3) in-person, yielding a *P* value of .96, while White participants had baseline scores of 61.05 (SD 2.33) for teleconsent and 59.92 (SD 3.31) for in-person, with a *P* value of .81. The *P* values across race categories ranged from .25 to .96, indicating a lack of statistical significance in the differences observed.

## Discussion

### Principal Findings

Our study provides important insights into how participants understand and make decisions during teleconsent sessions compared to traditional in-person IC visits. Notably, our results suggest that telehealth can match or even improve participants’ understanding of the consent process in certain situations. This study provides evidence that telehealth can effectively obtain IC for research studies in health. This challenges the common belief that in-person interactions are always better at facilitating informed consent. It also emphasizes the need to reevaluate consent methods in the evolving digital health landscape.

Findings from this study provide evidence that using telehealth to obtain IC is a viable method that allows participants to obtain similar levels of comprehension of the study consent form and make voluntary decisions as the in-person method. In some cases, we found that participants retained information better in the teleconsent group 30 days after the IC session compared to the in-person group. This supports previous findings suggesting that obtaining IC digitally can streamline the IC process compared to in-person methods [23].

The subgroup analysis suggests no significant differences in DMCI or QuIC scores across sexes, age groups, or races when comparing teleconsent and in-person methods. We found no significant differences in teleconsent participants’ comprehension levels of perceived voluntariness, trust, and decision self-efficacy from baseline to 30-day follow-up. The lack of significant differences provides evidence that using teleconsent among various populations can yield similar results to in-person IC efforts.

Our findings, aligned with prior studies, suggest the efficacy of telehealth in increasing accessibility and improving participant engagement in clinical research. For example, previous research has demonstrated that telehealth significantly reduces geographic barriers to seeking care [24]. Our findings build on this foundation by specifically targeting the comprehension aspect of the consent process, thereby filling an important gap in current scholarships.

However, teleconsent may also exacerbate health inequities among individuals and communities that lack access to digital tools or lack digital literacy [25,26]. Therefore, it is essential to carefully examine the use of teleconsent by conducting a needs assessment or feasibility study for the target audience to understand if teleconsent is an appropriate IC method.

The implications of our study findings are profound for future clinical research. As health care systems continue to adapt and incorporate telehealth, there is a clear opportunity to standardize teleconsent in many studies. Teleconsent has the potential to streamline participant enrollment and also foster a more inclusive environment where individuals who might otherwise face barriers to participation, such as those in remote areas or those with mobility challenges, can take part in important research without compromising their understanding of the process. Ultimately, this evolution in practice could improve participant understanding and engagement, advancing the goals of ethical, effective, and translational clinical research.

This study had limitations. It is a single-site study, and the diversity in some demographics was limited due to the difficulty of recruiting participants during a public health emergency. Also, we did not collect the duration of the consent session for each group, which would have added more evidence regarding differences in efficiencies between in-person and teleconsent methods. Future research should explore the long-term effects of teleconsent on participant engagement and retention, particularly among different populations. Studying how different technologies, such as smartphone apps, compare to traditional methods could provide valuable insights for optimizing telehealth strategies.

The findings of this study emphasize that study participants using teleconsent and in-person were able to comprehend and make voluntary decisions related to IC alike, that is, comprehension and voluntariness are not negatively impacted by this type of remote IC modality.

### Conclusions

This study aimed to assess the comprehension and decision-making effectiveness of IC processes conducted via telehealth compared to traditional in-person visits. Key findings revealed that telehealth maintains comparable levels of participant understanding and engagement, overcoming significant barriers such as geographic limitations and accessibility challenges. As health care increasingly adopts digital solutions, these findings highlight the potential of telehealth to enhance participant recruitment and retention in clinical research. This is particularly relevant for policy makers, who should integrate telehealth practices into regulatory frameworks to promote equitable access and improve health outcomes in diverse populations.

## Appendix

Multimedia Appendix 1 Survey Instrucments.

Multimedia Appendix 2 Comparison of baseline and follow-up scores for teleconsent and in-person surveys.

## Abbreviations

DMCI: Decision-Making Control Instrument
HIPAA: Health Insurance Portability and Accountability Act
IC: informed consent
QuIC: Quality of Informed Consent
SAHL-E: Short Assessment of Health Literacy-English
