# Supplementary material for: Effectiveness of Telehealth Versus In-Person Informed Consent: Randomized Study of Comprehension and Decision-Making
Source: J Med Internet Res. 2025 Mar 5;27:e63473. doi: 10.2196/63473 (PMC11923464; doi:10.2196/63473)
Supplement: Multimedia Appendix 1 [file jmir_v27i1e63473_app1.pdf]

Decision-Making Control Instrument

Sometime in the last ten days, you made a decision about enrolling yourself in a research study . We are interested in learning more about how you made this decision. Please respond to the following items and circle the one answer that best fits your opinion about this decision.

		**Strongly Disagree**	**Disagree**	**Somewhat Disagree**	**Somewhat Agree**	**Agree**	**Strongly Agree**
1	I was powerless in the face of this decision.	1	2	3	4	5	6
2	Someone took this decision away from me.	1	2	3	4	5	6
3	I made this decision.	1	2	3	4	5	6
4	I was passive in the face of this decision.	1	2	3	4	5	6
5	The decision about the protocol was inappropriately influenced by others.	1	2	3	4	5	6
6	I was not in control of this decision.	1	2	3	4	5	6
7	Others made this decision against my wishes.	1	2	3	4	5	6
8	I was not the one to choose.	1	2	3	4	5	6
9	The decision was up to me.	1	2	3	4	5	6

Research ID: __________

Research Participant Survey

PART A:

Instructions: Below you will find several statements about research studies. Thinking about the research study you just enrolled in, please read each statement carefully. Then tell us whether you agree with the statement, do not agree with the statement, or you are unsure about the statement by circling the appropriate response. Please respond to each statement as best as you can.
We are interested in your opinions.

A1.	When I signed the consent form, I knew I was agreeing to participate in the research study.	Disagree (-1)	Unsure (0)	Agree (1)
A2.	The main reason research studies are done is to improve the health of future patients.	Disagree (-1)	Unsure (0)	Agree (1)
A3.	I have been informed how long my participation in this research study is likely to last.	Disagree (-1)	Unsure (0)	Agree (1)
A4.	All the questionnaires and procedures in my research study visits are standard (routinely used) for X disease.	Disagree (-1)	Unsure (0)	Agree (1)
A5.	One of the main goals of this research study is to compare X disease patients with non-X disease patients to determine what might cause the disease.	Disagree (-1)	Unsure (0)	Agree (1)
A6.	Compared with standard treatments for my disease, my research study participation does not carry any additional risks or discomforts.	Disagree (-1)	Unsure (0)	Agree (1)
A7.	There may not be direct medical benefit to me from my participation in this research study.	Disagree (-1)	Unsure (0)	Agree (1)
A8.	By participating in this research study, I am helping the researchers learn information that may benefit future patients.	Disagree (-1)	Unsure (0)	Agree (1)
A9.	Because I am participating in a research study, it is possible that the study sponsor, various government agencies, or others who are not directly involved in my care could review my medical records.	Disagree (-1)	Unsure (0)	Agree (1)
A10.	My doctor did not offer me any alternatives besides participation in this research study.	Disagree (-1)	Unsure (0)	Agree (1)
A11.	The consent form I signed describes who will pay for treatment if I am injured or become ill as a result of participation in this research study.	Disagree (-1)	Unsure (0)	Agree (1)

A12.	The consent form I signed lists the name of the person (or persons) whom I should contact if I have any questions or concerns about the research study.	Disagree (-1)	Unsure (0)	Agree (1)
A13.	If I had not wanted to participate in this research study, I could have declined to sign the consent form.	Disagree (-1)	Unsure (0)	Agree (1)
A14.	I will have to remain in the research study even if I decide someday that I want to withdraw.	Disagree (-1)	Unsure (0)	Agree (1)

PART B:

Instructions: When you signed the consent form to participate in your research study, how well did you understand the following aspects of your research study? *If you didn't understand the item at all, please circle 1. If you understood it very well, please circle 5. If you understand it somewhat, please circle a number between 1 and 5.*

		I Didn't Understand this At All		→		I Understood this Very Well
B1.	How your participation in this research study may benefit future patients.	1	2	3	4	5
B2.	How long you will be in the research study.	1	2	3	4	5
B3.	What the researchers are trying to find out in the research study.	1	2	3	4	5
B4.	The questionnaires you will answer and procedures you will undergo.	1	2	3	4	5
B5.	The possible risks and discomforts of participating in the research study.	1	2	3	4	5
B6.	The possible benefits (or lack thereof) to you of participating in the research study.	1	2	3	4	5
B7.	The effect of the research study on the confidentiality of your medical records.	1	2	3	4	5
B8.	The alternatives to participation in the research study.	1	2	3	4	5
B9.	Who will pay for treatment if you are injured or become ill because of participation in this research study.	1	2	3	4	5
B10.	Whom you should contact if you have questions or concerns about the research study.	1	2	3	4	5
B11.	The fact that participation in the research study is voluntary.	1	2	3	4	5
B12.	Overall, how well did you understand your research study when you signed the consent form?	1	2	3	4	5

The 18 items of *SAHL-E*, ordered according to item difficulty (keys and distracters are listed in the same random order as in the field interview)

**Stem**	**Key or Distracter**		
1. kidney	__urine	__fever	__don't know
2. occupation	__work	__education	__don't know
3. medication	__instrument	__treatment	__don't know
4. nutrition	__healthy	__soda	__don't know
5. miscarriage	__loss	__marriage	__don't know
6. infection	__plant	__virus	__don't know
7. alcoholism	__addiction	__recreation	__don't know
8. pregnancy	__birth	__childhood	__don't know
9. seizure	__dizzy	__calm	__don't know
10. dose	__sleep	__amount	__don't know
11. hormones	__growth	__harmony	__don't know
12. abnormal	__different	__similar	__don't know
13. directed	__instruction	__decision	__don't know
14. nerves	__bored	__anxiety	__don't know
15. constipation	__blocked	__loose	__don't know
16. diagnosis	__evaluation	__recovery	__don't know
17. hemorrhoids	__veins	__heart	__don't know
18. syphilis	__contraception	__condom	__don't know
