# Supplementary material for: Effectiveness of Telehealth Versus In-Person Informed Consent: Randomized Study of Comprehension and Decision-Making
Source: J Med Internet Res. 2025 Mar 5;27:e63473. doi: 10.2196/63473 (PMC11923464; doi:10.2196/63473)
Supplement: Multimedia Appendix 2 [file jmir_v27i1e63473_app2.docx]

**Table S1.** Comparison of baseline and follow-up scores for teleconsent and in-person surveys.

| **Group** | **Survey** | **Baseline Average Score (SD)** | **Follow-up Average Score (SD)** | **P-value** |
| --- | --- | --- | --- | --- |
| **Teleconsent** | QuIC Part A:  Baseline vs. Follow up | 7.38 (1.29) | 7.84 (1.99) | 0.19 |
|  | QuIC Part B:  Baseline vs. Follow up | 53.88 (1.80) | 52.94 (3.03) | 0.07 |
|  | DMCI:  Baseline vs. Follow | 19.44 (1.12) | 19.41 (1.77) | 0.92 |
| **In-Person** | QuIC Part A:  Baseline vs. Follow up | 6.84 (2.46) | 7.03 (1.61) | 0.59 |
|  | QuIC Part B:  Baseline vs. Follow up | 53.19 (2.79) | 53.50 (2.68) | 0.56 |
|  | DMCI:  Baseline vs. Follow | 19.84 (2.31) | 19.09 (1.13) | 0.11 |
